# Neuropharmacological Treatment of Mental Dysfunction in Parkinson’s Disease

**DOI:** 10.1155/2006/138263

**Published:** 2006-05-11

**Authors:** Patrick McNamara, Raymon Durso

**Affiliations:** Department of NeurologyBoston University School of MedicineBoston VA Healthcare SystemBostonMAUSA

## Abstract

Many patients with Parkinson's Disease (PD) experience significant cognitive and mood impairment -even early in the course of the disease. These mental impairments are only partially responsive to levodopa treatment and are often as disabling as the motor impairment, particularly in mid and late stages of the disease. Investigators have recently begun a search for new agents that can effectively treat mental dysfunction of PD. Although there have been only a handful of properly controlled clinical trials of interventions targeted at amelioration of mental dysfunction in PD, progress has been made. Based on the available evidence, targeting catecholaminergic and cholinergic function may be an effective strategy for amelioration of cognitve, mood and psychiatric disturbances in PD.

## 1. Introduction

Parkinson disease (PD) is a progressive neurodegenerative disorder affecting approximately 1% of individuals older than 60 years. Cardinal motor features include resting tremor, rigidity, and bradykinesia. PD, however, also affects non-motor neurobehavioral functions and these features of PD may be just as disabling as the motor dysfunction. Options for treatment of non-motor manifestations of the disease, however, are not as well-investigated or well-developed as options for treatment of motor dysfunction of the disease.

Nevertheless, as neurobehavioral disorders of PD have become more widely appreciated some progress has been made in treatment of these disorders. In this paper we review that progress with an eye towards identifying promising new avenues for research in ameliorating mental dysfunction of PD.

## 2. Pathophysiology of PD consistent with mental dysfunction

The major neuropathologic findings in PD are a loss of pigmented dopaminergic neurons in the substantia nigra and the presence of Lewy bodies. Approximately 60–80% of dopaminergic neurons are lost before clinical symptoms of PD emerge. Loss of dopaminergic cells are most marked in the substantia nigra (SN) and in the ventral tegmental area or VTA [3]. These two subcortical dopaminergic sites give rise to two projection systems important for arousal, motor, affective and cognitive functioning. The nigrostriatal system, primarily implicated in motor functions, originates in the pars compacta of the SN and terminates in the striatum. The meso-limbic-cortical system contributes to cognitive and affective functioning. It originates in the VTA and terminates in the ventral striatum, limbic, and frontal systems. The degree of nigro-striatal impairment correlates with degree of motor impairment while VTA-mesocortical dopaminergic impairment correlates positively with the degree of affective and intellectual impairment [3,15,22,48–50,55] in affected individuals.

Although these dopaminergic systems are major contributors to mental dysfunction in PD, other neurotransmitter systems including the cholinergic, noradrenergic and serotonergic systems, are depleted in PD and likely contribute to mental dysfunction as well. In ad-

ISSN 0953-4180/06/$17.00 © 2006 – IOS Press and the authors. All rights reserved dition, Lewy body (LB) degeneration and Alzheimer-type changes have been noted in PD patients with mental dysfunction. These LB changes are found primarily in brainstem nuclei, (including the noradrenergic locus ceruleus and the serotonergic dorsal raphe nucleus), limbic structures, cholinergic forebrain structures and in cerebral cortex [13,23]. The cholinergic pathology in the basal forebrain structures and the LB-type degeneration in limbic and in cerebral cortex are likely contributors to cognitive and affective dysfunction of PD.

Lewy bodies are concentric, eosinophilic, cytoplasmic inclusions with peripheral halos and dense cores. The presence of Lewy bodies within pigmented neurons of the substantia nigra is characteristic, but not pathognomonic, of idiopathic PD as they are found in some cases of atypical parkinsonism and other disorders. The prevalence of incidental Lewy bodies increases with age. All Lewy bodies stain for alpha-synuclein and most also stain for ubiquitin. 'Lewy-body dementia' is distinguished from dementia of PD by a greater preponderance of visual hallucinations and marked fluctuations in attentional function throughout the day.

## 3. Prevention of neurobehavioral dysfunction not yet feasible

Given that most of the mental disorders of PD become severe or problematic in later stages of the disease the best strategy for treatment of these late stage complications of PD is prevention, rather than developing separate pharmacotherapeutic interventions for each symptom or disorder. There are several factors that have been implicated in the nigral cell death process in PD and that provide putative targets for neuroprotective therapy. These include oxidative stress, glutamate excitotoxicity, mitochondrial complex I deficit, inflammation, apoptosis, and growth factor deficit. Primary prevention of the disease, however, is currently not possible. Recent studies to slow, stop, or reverse neuronal death have shown some promising results although conclusive evidence is still pending [1,51,60].

Targeting single symptom complexes, of course, carries with it the danger of polypharmacy or of exacerbation of other symptom complexes not targeted by the drug. Although a drug, for example, may ameliorate late stage psychotic symptoms it may also increase motor dysfunction. Thus progress has been slow. Ideally, of course, one would want a drug that improves both motor and mental function in PD with little or no adverse side effects. It will be seen below that levodopa (LD) is the only agent that even approaches this ideal-at least in early to mid stages of the disease. In later stages where higher doses are involved, LD is associated with both motor and mental complications.

## 4. Frontal-basal ganglia circuits in Parkinson disease and pathophysiology of mental dysfunction in PD (see Fig. 1)

A series of neuroanatomical circuits or loops running between the basal ganglia and selected sites in the frontal lobes contribute to regulation of motor, motivational and mental functions in PD. Although details of specializations of each of the putative functional loops are problematic, the functional scheme is useful for organizing the mass of clinical and pathophysiologic data applicable to mental dysfunction in PD. The motor circuit, for example, modulates cortical output necessary for voluntary movement. When it is impaired, voluntary movement is impaired. The general functional scheme is this: Output from the motor (or motivational or cognitive or affective etc) circuit is directed through the internal segment of the globus pallidus (GPi) and the substantia nigra pars reticulata (SNr). This inhibitory output modulates the thalamocortical pathway itself and suppresses its actions (e.g. movement or thought depending on the circuit involved). To initiate movement, signals from the cerebral cortex are processed through the basal ganglia-thalamocortical motor circuit and return to the same area via a feedback pathway. When supplementary motor areas in the frontal lobes are released from sub-cortical inhibition, voluntary movement may occur. Similarly, a 'cognitive circuit' runs between the thalamus, globus pallidus, the dorsal caudate nucleus and thence to the dorsolateral prefrontal cortex. The 'motivational circuit' involves the ventromedial nucleus of the caudate and the lateral orbital frontal cortex and finally the limbic/emotional circuit involves the nucleus accumbens and the anterior cingulate cortex. Impairment of each of these specialized circuits in PD may partially contribute to characteristic types of mental dysfunction in PD with impairment of the dorsolateral prefrontal loop accounting for deficits in executive cognitive functions; impairment of the orbitofrontal loop contributing to apathy and related neurobehavioral signs and impairment in the limbic/cingulate circuit contributing to depression in PD.

Table 1
Current neuropharmacologic treatment options for mental dysfunction in PD

| Problem | Treatment | Comments |
|---|---|---|
| Mild cognitive deficits | Levodopa Cognitive-behavioral therapies (CBT) | Both levodopa and CBT have positive but transient effects on mild cognitive deficits of early PD |
| Dementia | Rivastigmine, donezepil, tacrine, galantamine | Most treatments target cholinergic system. Effects on motor systems are not as severe as expected/feared |
| Psychosis | Clozapine, quetiapine, olanzapine, risperidone, odensatron, rivastigmine, donepezil, tacrine, galantamine | Several of the atypical anti-psychotics also have positive effects on executive cognitive functions |
| Depression and anxiety | Trycyclics, MAO inhibitors, serotonin reuptake inhibitors, diazepam, ativan, clonzepam | It is still not clear that improving mood lastingly ameliorates cognitive deficits |

| Motor Circuit | Dorsolateral Circuit (cognition) | Orbital Circuit (motivation) | Cingulate Circuit (will/affect) |
|---|---|---|---|
| Supplementary, Premotor, and Motor Cortex | Dorsolateral Prefrontal Cortex | Lateral Orbitofrontal Cortex | Anterior Cingulate Cortex |
| ↓ | ↓ | ↓ | ↓ |
| Putamen | Caudate (Dorsolateral) | Caudate (Ventromedial) | Nucleus Accumbens |
| ↓ | ↓ | ↓ | ↓ |
| Globus Pallidus (Ventrolateral) | Globus Pallidus (Dorsomedial) | Globus Pallidus (Dorsomedial) | Globus Pallidus (Rostrolateral) |
| ↓ | ↓ | ↓ | ↓ |
| Thalamus (VA and MD) | Thalamus (VA and MD) | Thalamus (VA and MD) | Thalamus (MD) |

Fig. 1. Based ganglia- cortical loops affecting mental function in PD.

Two pathways exist within each basal ganglia-prefrontal circuit; a direct and an indirect pathway. In the direct pathway, outflow from the striatum directly inhibits GPi and SNr (i.e. the inhibitory modulating switch for the circuit itself). The indirect pathway comprises inhibitory connections between the striatum and the external segment of the globus pallidus (GPe) and the GPe and the subthalamic nucleus (STN). The subthalamic nucleus exerts an excitatory influence on the GPi and SNr. The GPi/SNr sends inhibitory output to the ventral lateral (VL) nucleus of the thalamus. Striatal neurons containing D1 receptors constitute the direct pathway and project to the GPi/SNr. Striatal neurons containing D2 receptors are part of the indirect pathway and project to the GPe.

To influence both motor and mental clinical symptoms of the disease various points in both the direct and indirect pathways have been targeted with pharmacologic, deep brain stimulation and surgical techniques. These targeted interventions are thought to work by altering inhibitory/excitatory balances along the direct and indirect pathways as well as along the basal ganglia – prefrontal loops.

## 5. Cognitive dysfunction in PD

Cognitive dysfunction is typically mild in early PD involving a generalized slowing of cognitive processing speed (bradyphrenia) and subtle deficits in so-called executive cognitive functions or ECFs [32,34, 36,57]. ECFs refer to such functions as planning, initiation, attention, monitoring and adjustment of non-routine and goal-directed behaviors. As the disease progresses, however, these ECF deficits become more severe and deficits in selected aspects of language and visuo-spatial functions become evident as well. Relative to age-matched controls, PD patients perform abnormally on tests of planning, switching attentional sets, verbal and semantic fluency, and selected complex visuo-spatial tasks [5,12,38,42,56,61]. Longitudinal studies of cognitive change in PD have repeatedly shown that performance on verbal and semantic fluency tasks are characteristic of the preclinical phase of dementia in PD and are significant predictors of dementia in PD [5,8,21,56,62].

## 6. Affective dysfunction in PD

Mood disturbances are also common in PD patients-even in the early stages of the disease. Symptoms of depression occur in up to 90% of PD patients, with about 40% developing the full clinical expression of depression. Similarly, anxiety disorders are seen in about 40% of PD patients. These mood disturbances have been shown to negatively impact quality of life, and daily functioning and may potentiate risk for cognitive decline as the disease progresses [30,40,46,52,56].

## 7. Risk factors for dementia in PD

About 30% of individuals with PD will develop a dementing illness as the disease progresses. Risk factors associated with the onset of a dementing illness in PD include age, late onset, lower educational level, disease duration, depression and ECF deficits (particularly deficits on verbal and semantic fluency) [8,20,43, 53,56].

Levodopa, still the most effective treatment of PD, is a precursor to both dopamine and norepinephrine (NE) and thus it behooves us to look briefly at the potential role of NE in mental disorders of PD.

## 8. Special role of norepineprine (NE) in ECF deficit in PD

Though not usually considered as a candidate in the explanation of cognitive deficits in PD, there is evidence that the role of NE is crucial. Post-mortem studies of cortical NE and MHPG consistently report decrements of these indices in PD patients with cognitive dysfunction. The locus ceruleus suffers severe cell loss in PD ranging from 21 to 93%, with an average of 63% compared with aged controls. LC cell loss correlates significantly with cognitive symptoms in PD – being more severe in patients with dementia than in those without dementia. LC damage results in severe loss of cortical and limbic NE innervation with a 40 to 78% decrease in NE, its metabolites and related enzymes in PD. These changes are most marked in patients with significant cognitive deficits. There are significantly decreased numbers of LC neurons *in dopa unresponsive PD patients* relative to dopa responsive patients. In clinical studies, Stern, Mayeux and Cote [54] found significant correlations between NE metabolite levels in PD patients with performance on reaction time tasks and continuous performance tasks that measure attention and vigilance.

Riekkinen et al. [47] reported strong correlations between measures of NE levels and attentional cognitive performance in PD. Bedard et al. [6] reported significant improvements on measures of attention and distractibility in a series of PD patients who were given the alpha-1 noradrenergic agonist naphtoxazine. Lemke [35] reported significant improvement on self- and objectively rated scores on a number of depression inventories in 16 PD patients with moderate to severe depression after 4 weeks of treatment with reboxetine, a NE re-uptake inhibitor. In short there is now clear evidence that NE dysfunction plays a role in production of cognitive and mood deficits of PD. Effects of levodopa on mental disorders of PD may therefore involve both of the major catecholaminergic systems projecting to forebrain sites. While we focus on potential dopaminergic sources of mental dysfunction in PD, the potential role of NE systems should be kept in mind as well.

## 9. Effects of levodopa on cognition in PD

Levodopa, coupled with a peripheral decarboxylase inhibitor (PDI), remains the gold standard treatment for PD. It provides the greatest motor benefit with the fewest adverse effects. Dopamine agonists may provide symptomatic benefit comparable to levodopa in early disease but may not in later stages. Nevertheless, both levodopa (LD) and the dopamine agonists have been shown to impact mental functions in PD. Generally speaking, cognitive performance improves on these dopaminergic agents at dose ranges sufficient to treat motor symptoms but negative effects have been observed as well. Interestingly, the positive effect is strongest for 'executive cognitive functions' (ECFs).

Both positive and negative effects on ECFs have been documented in the case of levodopa [9]. Gotham et al. [18] assessed the performance of PD patients on ECF tests that are known to be sensitive to prefrontal cortical dysfunction and found that verbal fluency was within normal limits while on levodopa but declined significantly (at least with the alternation task) while off levodopa. Patients were impaired on a 'set-switching task', the Wisconsin Card Sort test (WCST) both on and off levodopa. Kulisevsky et al. [28] found significant decrements in executive functions (including the Wisconsin card sort test) of a group of PD patients displaying major medication-related fluctuations in motor control compared with those showing a good response to therapy. The decrements occurred when off levodopa. Lange et al. [31,33] found that PD patients were dramatically impaired on 'frontal' or executive function tests (Tower of London task, set shifting, working memory, and spatial attention span) only when withdrawn from L-dopa medication. Performance on non-frontally-mediated tests such as visual memory tests was not impaired when patients were off levodopa.

Studies of attentional function in Parkinson's Disease suggest that forebrain dopamine, in addition to cortical norepinephrine, is crucially involved in attentional switching [31]. There is, for example, a well-established PD attentional switching deficit on 'category alternation paradigms' where the patients are required to generate names in one category for about a minute and then switch to another category [11]. PD patients find it difficult to generate an adequate number of names after switching categories. Administration of levodopa protects against this switching deficit for many patients who exhibit the deficit [31,33]. Dopaminergic activation may even support key components of the sentence comprehension system which depend on attentional processing and working memory [19]. McNamara et al. [39] studied 8 mild to severe PD patients who were tested both on and off levodopa using queries to test for working memory (WM) based comprehension of orally presented target sentences. They found that comprehension declined in the off state relative to the on state for all sentence types probed except 'direct object sentences' which required greater attentional/WM resources than the other sentence types.

Cools et al. [10] used PET to examine cortical and subcortical blood flow changes in relation to executive function performance in a group of PD patients both on and then off levodopa. Cools et al demonstrated that levodopa-related improvement in performance of the ECF tasks (Tower of London planning and spatial working memory) was associated with blood flow changes in right dorsolateral prefrontal cortex. Similarly, Knecht et al. [26] showed that healthy volunteers given 100 mg of LD per day exhibited more rapid and more accurate learning of verbal-visual associations than a group of controls given a placebo. Learning effects in this carefully controlled study could not be attributed to changes in arousal, autonomic function, motor response times, affect or response biases. LD clearly can affect high-level mental functions in humans and can improve performance on ECF tasks in patients with PD.

## 10. Effects of dopamine agonists on cognition in PD

While bromocriptine has repeatedly demonstrated mild but positive (enhanced) effects on measures of verbal fluency for patients with PD (and even patients with aphasia), the nonergoline agonists have not. In addition, more recently examined agonists such as pergolide and apomorphine have all demonstrated *negative* effects on verbal fluency [7,25,27]. Data on effects of ropinirole on fluency are not yet available, though it is not known why this is the case. Brusa et al. [7], for example, reported that treatment of patients with early PD with the D2 (and mild D3) DA agonist pramipexole (3.90 (1.19) mg three time per day), produced significant impairment on tests of short term verbal memory, and no improvement on tests of ECFs and verbal fluency. On the other hand, Kulisevsky et al. [29] noted a transient improvement on ECF tasks as well as verbal and visual memory for patients on pergolide, but the improvement on these cognitive tasks did not persist past 18 months of treatment.

## 11. Effects of cholinergic agents on cognition in PD

van Spaendonck et al. [58] reported that PD patients who were on anticholinergics evidenced poorer performance on tests of executive function than patients not on anti-cholinergics. In an open label trial and over the course of 26 weeks, Werber and Rabey [59] treated 11 demented patients with PD (PDD) with either tacrine (7 patients) or donepezil (4 patients) as add-on to their regular anti PD drugs. Cognitive assessment was performed at baseline and endpoint with the Mini-Mental-State-Examination (MMSE) and the Alzheimer's-Disease-Assessment-Scale (ADAS-cog). Mean ADAS-cog score but not the MMSE score across both drugs improved significantly by 3.2 points ($p < 0.012$) without a worsening in motor scores.

Aarsland et al. [59] using a double-blind randomized and placebo controlled design, studied effects of the cholinesterase inhibitor donepezil (5–10 mg per day) on Mini Mental State Exam scores and clinician plus caregiver-based ratings of change in 'activities of daily living' in 14 patients with moderate PD. After 10 weeks of treatment the mean MMSE score was increased by 2.1(2.7) points when on donepezil and virtually unchanged when on placebo. Similar donepezil-related enhancements were noted for clinician-caregiver ratings. Two of the 14 patients dropped out due to adverse side effects of the drug but the drug was well tolerated in the remaining patients. No worsening of motor scores/performance was noted while on the drug.

Similar findings were obtained in a series of open label trials of rivastigmine. Reading et al. [44] assessed 12 late stage PD patients. Three patients were able to tolerate 12 mg of rivastigmine per day; 5 patients 6 mg per day and 3 patients 9 mg per day and 1 patient only 3 mg per day. Rivastigmine was found to significantly reduce hallucinations and sleep disturbance as well as improve MMSE score with a mean gain score of 5 points. Nor did motor score change significantly. In an open-label trial, Giladi et al. [16] treated twenty-eight demented patients with PD for 26 weeks with rivastigmine (mean daily dose 7.2 $+/-$ 3.3 mg/day). Twenty patients completed 26 weeks of treatment and eight dropped out because of adverse side effects. The Unified Parkinson's Disease Rating Scale mental subscore improved significantly at week 26 while the motor score did not change. The mean ADAS-cog total score improved by 7.3 points at week 26. On the other hand, Richard et al. [45] reported a case of severe worsening of motor and mood function in a patient given a 3 mg oral dose of rivastigmine. More recently, an adequately powered placebo-controlled study [14], found that rivastigmine was associated with moderate improvements in dementia associated with Parkinson's disease but also with higher rates of nausea, vomiting, and tremor

Noting that cholinesterase inhibitors with additional nicotinic activity, such as galantamine, may be useful in PD patients with dementia (PDD) (since stimulation of nicotinic receptors may prevent the down-regulation that is likely to accompany cholinesterase inhibition and facilitate dopamine release in the striatum), Aarsland, Hutchison and Larsen [2], conducted an open label trial of galantamine in 16 patients with PDD. Improvement of global mental symptoms was noted in eight patients, whereas worsening was reported in four. Hallucinations improved in seven of the nine patients with hallucinations before treatment. Motor scores improved in six patients, but a mild worsening of tremor was noted in three.

In summary, a number of cholinergic agents have been tested in small-scale open label trials and with mid to late stage PD patients. While adverse side-effects were noted in all of these trials, adverse motor effects were not as severe as expected and there was a general trend in improvement in cognitive function while on the drug. Clearly, full-scale double blind placebo-controlled trials are needed to adequately evaluate these cholinergic agents in treatment of mental dysfunction in late stage PD.

## 12. Effects of other agents on cognition in PD

NMDA-type glutamate receptor antagonist drugs may reduce excitotoxic nerve damage and therefore protect against impaired cortical function in PD. Memantine, now under review for approval for use in the USA has demonstrated efficacy in small clinical trials in PD although the mechanism of action (neuroprotection vs. a symptomatic effect) is uncertain [4].

## 13. Treatment of psychiatric disturbances

As the disease progresses changes in mental status become more prominent with the appearance of a number of psychiatric symptoms that are very difficult to treat. At least 20% of PD patients manifest troublesome neuropsychiatric symptoms, particularly in mid to late stages of the disease.

The current management of psychosis in PD typically involves reducing dopaminergic dose levels and adding the use of atypical antipsychotic drugs. This frequently leads to a dilemma because a reduction in the dose or elimination of antiparkinsonian medications and/or the introduction of antipsychotic drugs can induce an unacceptable deterioration in parkinsonian motor symptoms. Patients are then left with the terrible choice of either 'losing their minds' or experiencing ever worsening motor fluctuations and deterioration. Fortunately, development of the new 'atypical anti-psychotics', particularly use of quietiapine are beginning to show some promising results with respect to this dilemma. Better success with these agents may be due, in part, to the fact that their action is primarily on serotonergic activity rather than D2 receptor antagonism.

## 14. Hallucinations and delusions

Hallucinations are thought to be experienced by as many as 33% of PD patients during the course of their illness. They are generally thought to be a complication of dopaminergic pharmacotherapy and when present limit drug therapy for motor disability. They are, furthermore, a significant risk factor for nursing home placement [17]. Hallucinations tend to occur in the visual rather than auditory modality and are frequently associated with vivid dreams [41]. Sleep disturbance appears more common in hallucinators than in nonhallucinators, but this insight has not yet been extensively studied.

With respect to pharmacotherapuetic options, compared to typical antipsychotics, atypical antipsychotics are less likely to produce a worsening of motor function in PD patients or of extrapyramidal symptoms (EPS) such as parkinsonism in non-PD psychotic patients.

In controlled studies, clozapine, the prototype of this new group of agents, has been shown to reduce psychotic symptoms in PD without aggravating parkinsonian motor symptoms. The use of this agent, however, has been limited by a 1 to 2% incidence of agranulocytosis and the requirement for frequent white blood cell count testing. Data on other atypical antipsychotics is mixed with reports of both benefit and motor deterioration in response to treatment with risperidone and olanzapine.

Quetiapine, another atypical agent available since 1998, has proven efficacy and a favorable EPS profile in schizophrenic and elderly patients. Clinical studies in schizophrenia indicate that the incidence of EPS across the full dosage range of quetiapine seems to be comparable to that of placebo. Furthermore, quetiapine has not been associated with agranulocytosis or other significant laboratory abnormalities. Open-label trials evaluating quetiapine in the elderly and in patients with PD with psychotic symptoms have reported good tolerability and statistically significant improvements for up to 12 months in measurements of psychotic symptoms on rating scales such as the Brief Psychiatric Rating Scale (BPRS) [24,37], but some patients required doses as high as 245 mg daily. Juncos et al. [24] found that the improvements in psychiatric symptoms on a relative low daily does of the drug (62.5 mg/day) in their cohort of PD patients were paralleled by significant improvements in immediate and delayed verbal memory, thus demonstrating that quietapine could improve cognitive function in PD along with reduction in psychotic symptoms.

## Acknowledgements

We thank Ms. Lena My Giang, for tracking down references and help formatting the paper. Preparation of this paper was supported, in part, by the Medical Research Service, Department of Veterans Affairs, Washington, DC.
